# 162. Duration of Antibiotics Through Care Transitions: A Quality Improvement Initiative

**DOI:** 10.1093/ofid/ofab466.364

**Published:** 2021-12-04

**Authors:** Caitlin Soto, Kate Dzintars, Kate Dzintars, Sara C Keller

**Affiliations:** 1 The Johns Hopkins Hosiptal, Baltimore, Maryland; 2 The Johns Hopkins Hospital, Baltimore, Maryland; 3 Johns Hopkins University School of Medicine, Baltimore, MD

## Abstract

**Background:**

Antibiotic resistance is increasing worldwide, largely driven by excessive antibiotic use. Antibiotic stewardship (AS) interventions have traditionally focused on acute care, long-term care, and ambulatory settings. However, as patients transition from one care setting to another, AS interventions should address antibiotic orders (agent, dose, duration) between the hospital and the home. The purpose of this study is to determine the appropriateness of a total course of antibiotics, including inpatient and outpatient prescriptions, to aid in prioritizing AS interventions.

**Methods:**

A single-center, retrospective study was performed to evaluate antibiotic duration for adult patients discharged from a large quaternary-care academic hospital. All antibiotic prescribing data, including pre-admission, during admission, and after hospital discharge, as well as information on indication, was collected from the electronic medical record. Descriptive statistics were used to summarize the data collected.

**Results:**

196 patients were included in the study. There were 100 instances of disagreement on antibiotic indication between the discharge summary and reviewer. However, 70% of patients were discharged on an appropriate antibiotic. The majority of patients (75%) were prescribed excess antibiotic days beyond guideline recommended total duration, and 68% of patients did not have appropriate duration of antibiotics post-discharge. Of those with excess duration, 31% were prescribed penicillins, 23% were prescribed cephalosporins, and 20% were prescribed trimethoprim/sulfamethoxazole. Excess antibiotic duration was associated most commonly with an unknown diagnosis (23%), a skin and soft tissue infection diagnosis (16%), and antibiotic prophylaxis (12%).

**Conclusion:**

The results of this study showed that patients were often prescribed excess antibiotics at discharge, and the total duration of antibiotics from pre-admission to post-discharge were prolonged beyond guideline-recommended duration. Understanding the total duration of antibiotic prescription, including post-discharge and pre-admission durations, is key in assessing risk from antibiotics and targeting AS interventions.

**Disclosures:**

**Kate Dzintars, PharmD**, Nothing to disclose

